# Personality traits and disorders among adult adhd patients: Do they vary between males and females?

**DOI:** 10.1192/j.eurpsy.2021.479

**Published:** 2021-08-13

**Authors:** I.F. Bracco, M. Boero, C. Mangiapane, F. Oliva

**Affiliations:** 1 Dipartimento Di Neuroscienze Rita Levi Montalcini, Università degli Studi di Torino, Torino, Italy; 2 Dipartimento Di Scienze Cliniche E Biologiche, Università di Torino, Torino, Italy

**Keywords:** ADHD, personality disorder, Gender differences

## Abstract

**Introduction:**

Patients with Attention Deficit/Hyperactivity Disorder (ADHD) have shown an increased risk of developing a DSM Cluster B (i.e., Borderline, OR=13.16; Antisocial, OR=3.03; Narcissistic, OR=8.69) and DSM Avoidant Personality Disorder (PD; OR=9.77; Miller et al., 2008). Although different comorbidities affect males and females with ADHD (Kooij et al., 2013), gender differences in personality traits and disorders have not yet been investigated.

**Objectives:**

To describe gender differences in personality traits and disorders among a sample of adult outpatients with ADHD.

**Methods:**

A consecutive sample of DSM-5 ADHD outpatients was recruited at the Adult ADHD Center of the “San Luigi” University Hospital (Orbassano (TO), Italy) between Jan 2017 and Jan 2018. Patients’ personality was assessed by Millon Clinical Multiaxial Inventory (MCMI-III; Zennaro et al, 2008).

**Results:**

The study sample consisted of 82 males and 31 females. Sixty percent of men vs. 77% of women had a personality disorder (

**Conclusions:**

Women with ADHD showed a higher frequency of personality disorders and higher rate of Masochistic PD than men. Moreover, the two most important clusters detected in women included severe personality components (i.e., Borderline and Paranoid) when compared with men. Further studies on larger samples should be conducted to confirm more severe personality profiles in women than in men.

**Disclosure:**

No significant relationships.

